# Effects of hydrothermal pretreatment on methane potential of anaerobic digestion sludge cake of cattle manure containing sawdust as bedding materials

**DOI:** 10.5713/ab.22.0434

**Published:** 2023-02-26

**Authors:** Jun-Hyeong Lee, Chang-Hyun Kim, Young-Man Yoon

**Affiliations:** 1 Biogas Research Center, Hankyong National University, Anseong 17579, Korea; 2 Department of Plant Life & Environmental Science, Hankyong National University, Anseong 17579, Korea; 3 Department of Animal Resource Science, Hankyong National university, Anseong 17579 Korea

**Keywords:** Anaerobic Digestion, Biochemical Methane Potential, Cattle Manure, Hydrothermal Pretreatment, Organic Matter Solubilization

## Abstract

**Objective:**

The purpose of this study was to analyze the effect of the hydrothermal pretreatment of anaerobic digestion sludge cake (ADSC) of cattle manure on the solubilization of organic matter and the methane yield to improve the anaerobic digestion efficiency of cattle manure collected from the sawdust pens of cattle.

**Methods:**

Anaerobic digestion sludge cake of cattle manure was thermally pretreated at 160°C, 180°C, 200°C, and 220°C by a hydrothermal pressure reactor, and the biochemical methane potential of ADSC hydrolysate was analyzed. Methane yield recovered by the hydrothermal pretreatment of ADCS was estimated based on mass balance.

**Results:**

The chemical oxygen demand solubilization degree (COD_s_) of the hydrothermal hydrolysate increased to 63.56%, 67.13%, 70.07%, and 66.14% at the hydrothermal reaction temperatures of 160°C, 180°C, 200°C, and 220°C, respectively. Considering the volatile solids content obtained after the hydrothermal pretreatment, the methane of 10.2 Nm^3^/ton-ADSC was recovered from ADSC of 1.0 ton, and methane yields of ADSC hydrolysate increased to 15.6, 18.0, 17.4, and 17.2 Nm^3^/ton-ADSC.

**Conclusion:**

Therefore, the optimal hydrothermal reaction temperature that yielded the maximum methane yield was 180°C based on mass balance, and the methane yield from cattle manure containing sawdust was improved by the hydrothermal pretreatment of ADSC.

## INTRODUCTION

The total amount of livestock manure generated in Korea in 2019 was about 153,220 ton/d of which the amount of cattle manure was reported to be about 62,608 ton/d. In general, cattle (beef and dairy cattle) breeding facilities mainly adopt sawdust feedlots in which sawdust is adopted as the bedding material. Most of the livestock manure generated in the solid phase at the sawdust feedlot is composted and used as a fertilizer resource for agricultural land. Therefore, in the case of concentrated cattle breeding areas, the existence of non-point pollution sources affecting the water system remains a serious concern owing to the outflow of excessive nitrogen and phosphorus resulting from the application of cattle manure as compost to farmland. In particular, in Korea, the 2050 carbon-neutral policy, which requires national greenhouse gas emission to be net zero by 2050, has been established, and interest in reducing greenhouse gas emissions by converting livestock manure into bioenergy is increasing. Quantitatively, the energy potential of the biomass that can be converted and used as bioenergy was assessed as 760,032 TOE/yr for cattle manure, 314,493 TOE/yr for sewage sludge, 411,656 TOE/yr for food waste, and 196,320 TOE/yr for pig slurry, respectively. Considering that the bioenergy potential of cattle manure was evaluated to be the highest, a great need exists to promote the conversion of cattle manure into bioenergy as part of future plans to generate bioenergy from livestock manure.

In Korea, cattle manure discharged from cattle breeding facilities consists of a mixture of livestock manure and the sawdust used as bedding material. In particular, sawdust is lignocellulosic biomass and contains a large amount of lignin, which is biologically difficult to decompose and is poorly decomposed during anaerobic digestion. In addition, cattle manure hydrolyzes slowly during the anaerobic digestion process. This decreases the anaerobic decomposition efficiency of organic matter and increases the generation of anaerobic digestion sludge, which impedes the economic feasibility of biogas facilities for processing cattle manure [1]. Especially, anaerobic digestion sludge cake (ADSC) contains the moisture content above 80%, it is disposed after incineration due to the prohibition of direct landfill and ocean disposal in Korea. Nowadays, the interest in energy conversion of sludge waste is increasing due to high sludge disposal costs and limited alternative disposal methods. However, ADSC of cattle manure is characterized by a high solid content that is composed of lignocellulosic matter. Therefore, it is difficult to be fed into conventional anaerobic digesters. Therefore, recently, various technologies to enhance the hydrolysis efficiency, including physical, chemical, and biological pretreatment, have been studied to improve the anaerobic digestion efficiency of cattle manure [2]. Hydrothermal pretreatment promotes the hydrolysis of difficult-to-decompose organic substances based on thermochemical reactions [3,4]. Hydrothermal pretreatment can promote the hydrolysis of organic matter by treating organic material with a moisture content of 70% to 80% with pressurized hot water at 200°C to 300°C. The hydrothermal reaction proceeds via complex mechanisms such as dehydration, carboxylation, decarboxylation, and condensation to hydrolyze and carbonize organic matter. As a result of these reaction mechanisms, hydrothermal pretreatment has been reported to improve the efficiency of solid-liquid separation by increasing the dehydration properties of the hydrothermal hydrolysate and the hydrolysis of organic matter [4,5]. Therefore, hydrothermal pretreatment increases the methane production rate by accelerating the hydrolysis of organic matter. This reaction characteristic shortens the hydraulic retention time of the anaerobic digester, thereby reducing its effective volume [6]. The technical characteristics of this hydrothermal pretreatment can effectively shorten the operating time of the process by utilizing the by-products (e.g., anaerobic digestion sludge) of the anaerobic digestion process when applied to conventional anaerobic digestion technology [7,8]. Furthermore, the application of hydrothermal pretreatment technology to the anaerobic digestion of cattle manure containing a large amount of cellulosic material reportedly increases the bioenergy recovery efficiency by 48.2% to 60.0% [9–11]. However, despite these technical advantages, the application of hydrothermal pretreatment technology to discharged cattle manure containing sawdust is uncommon in Korea. Therefore, this study aimed to improve the anaerobic digestion efficiency of cattle manure mixed with sawdust by analyzing the effect of hydrothermal pretreatment on the solubilization of organic matter and the potential increase in the amount of methane produced from the ADSC of the cattle manure. Experimentally, the purpose of this study was to derive the optimal hydrothermal pretreatment temperature to improve the anaerobic digestion efficiency.

## MATERIALS AND METHODS

### Materials

Cattle manure was collected from the feedlot of beef cattle farmhouse, and the feedstock for anaerobic digestion was prepared with the mixture of cattle manure and pig slurry for moisture control. Then, the moisture characteristics of cattle manure give difficulty at the mixing of wet type anaerobic digester. Therefore, pig slurry was used as the moisture regulator for the improvement of mixing efficiency of anaerobic digester. Thereafter, the anaerobic digestion sludge of cattle manure was collected from a pilot scale PFR (Plug and flow reactor) type anaerobic digester (effective volume = 100 L) that was operated as the hydraulic retention time of 30 days in the mesophilic condition (38°C). Then, the collected anaerobic digestion sludge was centrifuged at 4,000 rpm for 20 min, thus preparing the ADSC.

### Hydrothermal pretreatment

A batch-type hydrothermal reactor was designed and developed for the hydrothermal pretreatment of ADSC. The hydrothermal reactor was a closed system with no potential heat loss via vaporization and condensation. The designed hydrothermal reactor had a working volume of 1.5 kg and was equipped with an electric heater (a heating coil), a temperature sensor, and a pressure gauge. The temperature sensor and pressure gauge were inserted into the reactor to monitor the inner temperature and saturated vapor pressure during the hydrothermal reaction. The sludge cake (1.5 kg) was placed directly in the reactor without additional processing water and the reactor was sealed with an airtight sealant for the hydrothermal reaction test. The temperature settings were 160°C, 180°C, 200°C, and 220°C. When the temperature in the reactor reached each of these settings, isothermic conditions were maintained for 60 min. The inner vapor pressures corresponding to these temperatures were 0.85 MPa at 160°C, 1.18 MPa at 180°C, 1.78 MPa at 200°C, and 2.51 MPa at 220°C. The hydrothermal reactor was cooled to room temperature at the end of the hydrothermal reaction using a chiller, whereupon the hydrothermal hydrolysates were recovered from the reactor.

### Methane production potential

The theoretical methane potential (B_th_) was calculated stoichiometrically using Boyle’s equation based on the elemental analysis results of the samples (Equation 1 and 2) [12].


(1) $$\begin{array}{l} C_{a}H_{b}O_{c}N_{d}S_{e}+\left(a-\frac{b}{4}-\frac{c}{2}+\frac{3d}{4}+\frac{e}{2}\right)H_{2}O\to \\ \left(\frac{a}{2}+\frac{b}{8}-\frac{c}{4}-\frac{3d}{8}-\frac{e}{4}\right){CH}_{4}+\left(\frac{a}{2}-\frac{b}{8}+\frac{c}{4}+\frac{3d}{8}+\frac{e}{4}\right){CO}_{2}\\ +{dNH}_{3}+{eH}_{2}S \end{array}$$

(2) $$\begin{array}{l} B_{th}({Nm}^{3}-{CH}_{4}/kg-{VS}_{added})\\ =22.4\times (\frac{\left(4a+b-2c-3d-2e)/8\right)}{12a+b+16c+14d+32e}) \end{array}$$

The ultimate methane potential (B_u_) was assessed by the biochemical methane potential assay [13]. To assess the biochemical methane potential of the hydrothermal hydrolysate, a batch-type anaerobic reactor was operated under mesophilic conditions (38°C). The anaerobic inoculum was collected from a farm-scale anaerobic digester located in Icheon, Korea. The chemical properties of the inoculum are provided in Table 1.

The inoculum for the biochemical methane potential assay of the hydrothermal hydrolysate was kept under mesophilic conditions at 38°C for one week to remove any remaining biodegradable fraction. The substrate to inoculum ratio in all anaerobic batch reactors was equal to 0.5 (g-VS_substrate_/g-VS_inoculum_). The working volume for anaerobic batch fermentation was 80 mL of a 160 mL serum bottle. The headspace of the serum bottle was filled with N_2_ gas and sealed with a butyl rubber stopper. The anaerobic batch reactors for each sample and blank were incubated for up to 90 days in the convection incubator and manually mixed each day during the fermentation period. Then, the anaerobic batch reactors for each sample and blank were performed in three replicates. The biochemical methane potential was calculated based on the volatile solid (VS) content. The biochemical methane potentials of the samples were corrected using the blank value, and calibrated under standard temperature and pressure (STP) conditions (0°C, 1 atm). The modified Gompertz model (Equation 3) [14] and the parallel first-order kinetic model (Equation 4) were employed to interpret the progress of cumulative methane production. This enabled the cumulative methane production data to be optimized using these equations [15]. Especially, the modified Gompertz model was applied for the estimation of lag phase time and maximum methane production rate, and the parallel first-order kinetic model was used the estimation of organic fractionation composing of ADSC.


(3) $$M_{t}=P\times \text{exp}\;\left[-\text{exp}\left(\frac{R_{m}\times e}{P}[\lambda -t]+1\right)\right]$$

where M_t_ is the cumulative methane production (mL), t is the anaerobic fermentation time (days), P is the final methane production (mL), e is the exp (1), R_m_ is the maximum methane production rate (mL/d), and λ represents the lag growth phase time (days).


(4) $$B_{t}=B_{max}[1-f_{e}e^{-k_{1}t}-(1-f_{e})e^{-k_{2}t}]$$

where B_t_ (mL) is the amount of methane production at time t, B_max_ (mL) is the ultimate amount of methane production, f_e_ (g/g) is the organic distribution constant for the two first-order kinetic models, and k_1_ and k_2_ are the kinetic constants in the parallel first-order kinetics. The cumulative methane production curves of the hydrothermal hydrolysates were optimized with SigmaPlot (SigmaPlot Version 12.5; Systat Software Inc., Cary, NC, USA) using the modified Gompertz and parallel first-order kinetic models, respectively. The degree of optimization by the two mathematical models was evaluated by the root mean square deviation (RMSD) (Equation 5).


(5) $$RMSD=\sqrt{\frac{1}{n}\sum_{t=1}^{n}{\left[B(t)-B^{\prime }(t)\right]}^{2}}$$

### Estimation of fraction of organic matter

The parallel first-order kinetic model (Equation 4) considers that the degradation of organic matter sequentially occurs in two stages. In addition, f_e_ distributes the characteristics of the two types of substrates with different reaction rates under anaerobic conditions, and k_1_ and k_2_ indicate the first-order kinetics constants for the first and second organic degradation stages, respectively. In this study, based on the characteristics of the parallel first-order kinetic model, the characteristics of the organic material composition of the substrate were estimated from the analysis results of the rate of the decomposition reaction of the organic material. The total volatile solids (VS_T_) of the substrate were assumed to consist of a biodegradable volatile solid fraction (VS_B_) and a non-biodegradable volatile solid fraction (VS_NB_), as in Equation 6. The biodegradable organic fraction was defined as consisting of easily biodegradable volatile solids (VS_e_) that were readily decomposed in the early stage of anaerobic digestion and persistently biodegradable volatile solids (VS_p_) that were slowly decomposed owing to their resistance to decomposition as in Equation 7 [7]. Then, the composition fraction of VS_e_ and VS_p_ can be estimated by the f_e_ (the organic distribution constant for the two first-order kinetics, g/g) as in Equation 8.


(6) $${VT}_{T}={VS}_{B}+{VS}_{NB}$$

where VS_T_ is the total volatile solid content (g), VS_B_ is the biodegradable volatile solids content (g), and VS_NB_ is the non-biodegradable volatile solids content (g).

In Equation 6, VS_B_ may be considered to be represented by B_u_/B_th_. Then, Equation 7 is induced.


(7) $${VS}_{B}={VS}_{T}(1-\frac{B_{u}}{B_{th}})$$

where B_u_ is the ultimate methane potential (Nm^3^-CH_4_/kg-VS_added_) and B_th_ is the theoretical methane potential (Nm^3^-CH_4_/kg-VS_added_).


(8) $${VS}_{B}={VS}_{e}+{VS}_{p}=f_{e}\times {VS}_{B}+(1-f_{e}){VS}_{B}$$

where VS_e_ is the easily biodegradable volatile solid content (%, w/w), VS_p_ is the persistently biodegradable volatile solid content (%, w/w), and f_e_ is the organic distribution constant for the two first-order kinetics models (VS_e_/VS_B_, g/g).

### Analysis

The total solids (TS), VS, pH, chemical oxygen demand (COD_Cr_), soluble chemical oxygen demand (SCOD_Cr_), total kjeldahl nitrogen (TKN), ammonium nitrogen (NH_4_^+^-N), and alkalinity were determined based on standard methods [16]. The total volatile fatty acids (TVFAs) were measured using a gas chromatograph (GC2010; Shimadzu Scientific Instruments, Inc., Columbia, MD, USA) equipped with a flame ionization detector with an automatic sampler. This chemical analysis was performed in three replicates. The elemental composition (C, H, N, O, S) was determined using an element analyzer (EA1108; Thermo Finnigan LLC, San Jose, CA, USA) and the COD_Cr_ solubilization degree (CODs) of the hydrothermal hydrolysate was calculated by Equation 9, where COD_s_ represents the COD_Cr_ solubilization degree of the hydrothermal hydrolysate, SCOD_Cr-hydrolysate_ represents the SCOD_Cr_ of the hydrothermal hydrolysate, and SCOD_Cr-ADSC_ represents the SCOD_Cr_ of ADSC.


(9) $${COD}_{S}=\frac{\left(S{COD}_{Cr-hydrolysate}-S{COD}_{Cr-ADSC}\right)}{S{COD}_{Cr-hydrlysate}}\times 100$$

In the anaerobic batch reactor experiment, the total gas production was measured daily for the first five days and then every two or three days. The gas that was produced displaced an acidified brine solution in a burette and the volume of displaced solution was recorded after correcting for atmospheric pressure [17]. The CH_4_ and CO_2_ concentrations in the gas samples were determined using a gas chromatograph (Clarus 680; PerkinElmer, Inc., Waltham, MA, USA) equipped with a thermal conductivity detector and a HayeSepQ packed column (CRS Inc., Louisville, KY, USA). The column was operated with helium carrier gas at a flow rate of 5 mL/min. The temperatures of the injector, oven, and detector were set to 150°C, 90°C, and 150°C, respectively [14].

### Statistical analysis

The tables in this article present the mean values and standard deviations of the data obtained from the experiments. The statistical analysis of the results of this experiment was analyzed using the general linear model procedure of the SAS program package (SAS ver. 9.4; SAS instrument Inc., Cary, NC, USA), and the significant difference (p<0.05) of the mean between treatments was tested through Duncan’s multiple range test.

## RESULTS AND DISCUSSION

### Physicochemical properties of ADSC and ADSC hydrolysates

Table 2 presents the elemental analysis results and theoretical methane potentials of the hydrothermal hydrolysate obtained by the hydrothermal pretreatment of ADSC at 160°C, 180°C, 200°C, and 220°C and the anaerobically digested sludge cake (ADSC) of the cattle feedlot manure. The carbon (C) content of the ADSC was 34.0%, and that of the hydrothermal hydrolysate at the reaction temperatures of 160°C, 180°C, 200°C, and 220°C was 37.5%, 37.2%, 36.4%, and 35.2%, respectively. Based on the results of the elemental analysis, the theoretical methane potential was stoichiometrically calculated according to Boyle’s equations (Equation 1 and 2), and the theoretical methane potential (B_th_) of ADSC was 0.425 Nm^3^/kg-VS_added_. The B_th_ of the hydrothermal hydrolysates were 0.478, 0.462, 0.496, and 0.466 Nm^3^/kg-VS_added_ at the hydrothermal reaction temperatures of 160°C, 180°C, 200°C, and 220°C, respectively. Therefore, the hydrothermal pretreatment of ADSC increased the carbon content and theoretical methane potential of the hydrolysate, with the highest theoretical methane potential corresponding to the reaction temperature of 200°C for hydrothermal pretreatment. Table 3 presents the physicochemical analysis of ADSC and hydrothermal hydrolysate. The TS and VS contents of the hydrothermal hydrolysate have the lowest values of 203,799 and 140,056 mg/kg, respectively, at the reaction temperature of 200°C. The value of SCOD_Cr_ was 39,595, 43,900, 48,215, and 42,620 mg/L for the 160°C, 180°C, 200°C, and 220°C hydrothermal hydrolysates, respectively. The solubilization degree of COD (COD_s_) increased to 63.56%, 67.13%, 70.07%, and 66.14% for the hydrothermal hydrolysates at 160°C, 180°C, 200°C, and 220°C compared to ADSC, respectively. Generally, hydrothermal pretreatment using pig manure, cattle manure, chicken manure, etc. reportedly differs in terms of the solubilization degree of organic matter and the effect of temperature, depending on the characteristics of the raw material [18–20]. In addition, as the hydrothermal pretreatment entails the hydrolysis and carbonization reactions of the organic matter, the amount of elemental carbon and the theoretical methane potential increased, as reported previously [21]. Therefore, the hydrothermal hydrolysate can be easily converted to methane in an anaerobic digester [22].

### Biochemical methane potential assay

**Figure 1:**
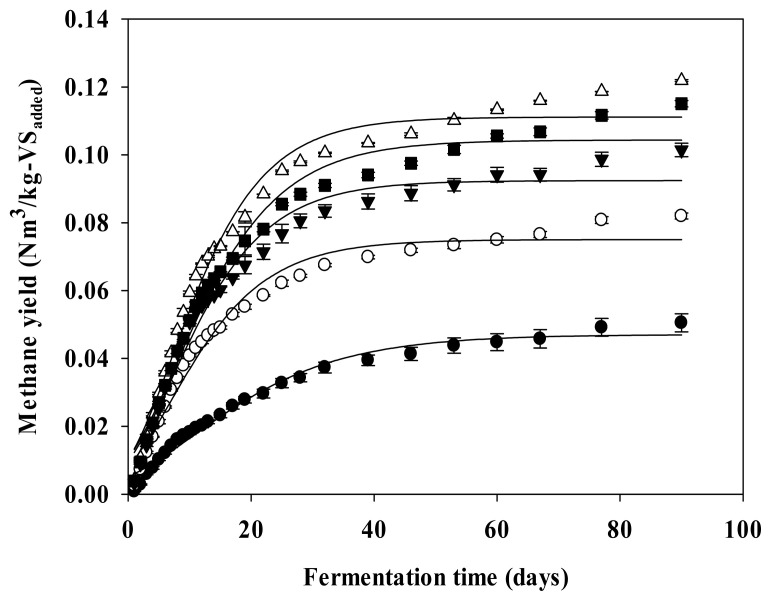
Methane yield curves optimized by the modified Gompertz model in the hydrothermal pretreatment of ADSC (Vertical bar means standard error, n = 3). The filled cycle (●) shape represents ADSC, blank circle (○) shape represents hydrolysate (160°C), filled inverted triangle (▼) shape represents hydrolysate (180°C), blank triangle (Δ) shape represents hydrolysate (200°C), and filled quadrangle (■) shape represents hydrolysate (220°C). ADSC, anerobic digestion sludge cake.

**Figure 2:**
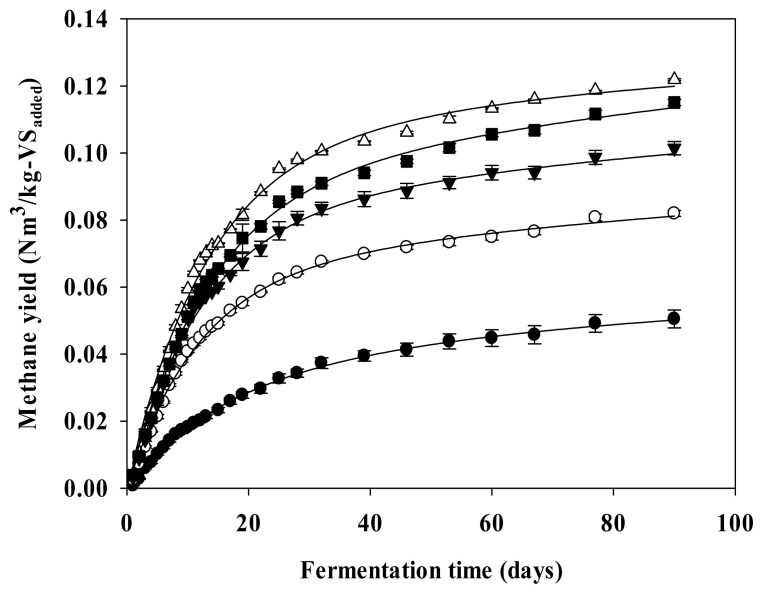
Methane yield curves optimized by the Parallel first-order kinetic model in the hydrothermal pretreatment of ADSC (Vertical bar means standard error, n = 3). The filled cycle (●) shape represents ADSC, blank circle (○) shape represents hydrolysate (160°C), filled inverted triangle (▼) shape represents hydrolysate (180°C), blank triangle (Δ) shape represents hydrolysate (200°C), and filled quadrangle (■) shape represents hydrolysate (220°C). ADSC, anerobic digestion sludge cake.

Figure 1 shows the cumulative methane production curve of the ADSC hydrothermal hydrolysate optimized with the modified Gompertz model (Equation 3). The parameters obtained by the modified Gompertz model are listed in Table 4. The methane potential (B_u_-G) of the ADSC hydrothermal hydrolysate, estimated with the modified Gompertz model, was 0.075, 0.092, 0.112, and 0.104 Nm^3^/kg-VS_added_ at the hydrothermal pretreatment reaction temperatures of 160°C, 180°C, 200°C, and 220°C, respectively. Moreover, the methane yields increased by 59.57%, 95.74%, 138.30%, and 121.28% relative to the B_u_-G of ADSC (0.047 Nm^3^/kg-VS_added_), respectively, and the hydrothermal pretreatment reaction temperature of 200°C yielded the highest amount of methane. The maximum methane production rates (R_m_) of the ADSC hydrothermal hydrolysate were 3.9, 4.8, 5.8, and 5.3 mL/d, respectively. In comparison with the R_m_ (2.6 mL/d) of ADSC, the R_m_ of the ADSC hydrothermal hydrolysate increased to 50.00%, 88.46%, 123.08%, and 103.85% at hydrothermal pretreatment reaction temperatures of 160°C, 180°C, 200°C, 220°C, respectively. The lag growth phase time (λ) of ADSC was 2.7 days and the lag growth phase times (λ) of the ADSC hydrothermal hydrolysate were 1.7, 1.6, 1.1, and 1.6 days at the hydrothermal pretreatment reaction temperatures of 160°C, 180°C, 200°C, and 220°C, respectively. Thus, the hydrothermal pretreatment time of ADSC was shortened by 37.04%, 40.74%, 59.26%, and 40.74% compared to λ of ADSC, respectively. Figure 2 shows the cumulative methane production curve of the ADSC hydrothermal hydrolysate optimized with the parallel first-order kinetic model (Equation 4). The parameters determined with this model are listed in Table 5. The methane potential (B_u_-P) of the ADSC hydrothermal hydrolysate estimated with the parallel first-order kinetic model, was 0.086, 0.105, 0.124, and 0.118 Nm^3^/kg-VS_added_ at the hydrothermal pretreatment reaction temperatures of 160°C, 180°C, 200°C, and 220°C, respectively. Thus, the hydrothermal pretreatment reaction temperature of 200°C yielded the largest amount of methane. A comparison of the performance of the modified Gompertz model and the parallel first-order kinetic model with respect to optimizing the cumulative methane production curve of the ADCS hydrothermal hydrolysate, respectively, revealed that the RMSD of the former model was in the range of 0.006 to 0.010, and that of the latter model ranged from 0.001 to 0.002. Therefore, the parallel first-order kinetic model was more suitable for the analysis of the cumulative methane production curve of the ADSC hydrothermal hydrolysate containing persistently biodegradable VS.

### Changes of VS fractionation and methane production

**Figure 3:**
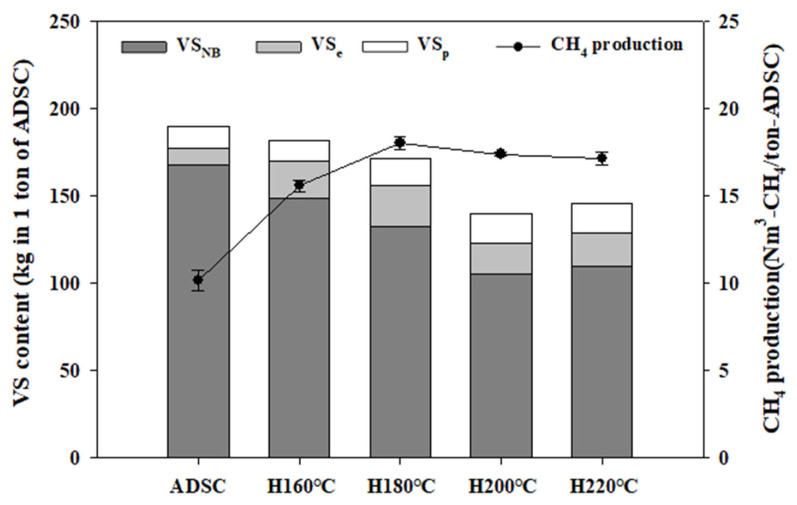
Methane yield and VS fractionation in the hydrothermal pretreatment of ADSC (Vertical bar means standard error, n = 3). The ADSC indicates anaerobic digestion sludge cake, and H160°C, H180°C, H200°C, and H220°C indicate hydrothermal pre-treatment reaction temperature. The VS_BN_ means non-biodegradable volatile solid fraction, the VS_e_ means easily biodegradable volatile solid fraction, the VS_p_ means persistently biodegradable volatile solid fraction, and the filled cycle (●) shape represents CH_4_ production.

The organic distribution constant (f_e_), which indicates the distribution of the easily biodegradable volatile solids (VS_e_) and persistently biodegradable volatile solids (VS_p_), was estimated to be 0.066 for the ADSC. In comparison, the f_e_ of the ADSC hydrothermal hydrolysate was 0.641, 0.651, 0.669, and 0.540 at the hydrothermal pretreatment reaction temperatures of 160°C, 180°C, 200°C, and 220°C, respectively. The organic matter (VS) contained in the ADSC was characterized as consisting of 11.9% biodegradable volatile solids (VS_B_) and 88.1% non-biodegradable volatile solids (VS_NB_). More specifically, the VS_B_ fraction of ADSC was estimated to be composed of VS_e_ of 5.5% and VS_p_ of 6.3%. The VS_B_ fraction of the ADSC hydrothermal hydrolysate increased to 17.9%, 23.3%, 26.7%, and 23.7% at hydrothermal reaction temperatures of 160°C, 180°C, 200°C, and 220°C, respectively, whereas VS_NB_ decreased to 82.1%, 76.7%, 73.3%, and 76.3%, respectively. Regarding the VS_B_ fraction of the ADSC hydrothermal hydrolysate, 11.5%, 15.2%, 17.8%, and 12.8% were estimated to be VS_e_, and 6.5%, 8.1%, 8.8%, and 10.9% to be VS_p_ at the hydrothermal reaction temperatures of 160°C, 180°C, 200°C, and 220°C, respectively. As aforementioned, the hydrothermal pretreatment reaction temperature of 200°C yielded the largest amount of methane. However, considering the VS content obtained after the hydrothermal pretreatment, VS obtained from the ADSC of 1ton was 189.5 kg, and VS obtained from ADSC hydrolysate were 181.6, 171.8, 140.1, and 145.9 kg at the hydrothermal reaction temperatures of 160°C, 180°C, 200°C, and 220°C, respectively. Then, the methane of 10.2 Nm^3^/ton-ADSC was recovered from ADSC of 1.0 ton, and methane yields of ADSC hydrolysate increased to 15.6, 18.0, 17.4, and 17.2 Nm^3^/ton-ADSC (Figure 3). Therefore, the optimal hydrothermal reaction temperature that yielded the maximum methane yield was 180°C based on mass balance.

Hydrothermal pretreatment was shown to be an efficient method for hydrolyzing cattle manure containing difficult-to-decompose organic matter. However, hydrothermal pretreatment has been reported to lead to different degrees of solubilization of organic substances depending on the constituents of the raw materials, reaction temperature, and reaction time [21,23,24]. In this study, a hydrothermal reaction temperature of 200°C was determined to be the optimal temperature at which the methane yield is maximized. However, as the hydrothermal reaction temperature increased, the VS_e_ and VS_p_ fractions increased simultaneously with the VS_p_ content (persistently biodegradable) changing most significantly at the hydrothermal reaction temperature of 200°C. Marin-Batista et al [10] reported a methane yield of 0.111 Nm^3^/kg-VS_added_ from the anaerobic digestion of cattle manure and reported yields of 0.294, 0.235, and 0.080 Nm^3^/kg-VS_added_ from the hydrothermal hydrolysates at hydrothermal reaction temperatures of 170°C, 200°C, and 230°C, respectively. In addition, Kim et al [25] reported methane potentials of 0.197, 0.231, 0.221, and 0.200 Nm^3^/kg-VS_added_ for pig sludge hydrothermally pretreated at 200°C, 220°C, 250°C, and 270°C, respectively. These results are consistent with the results of our study in that the maximum methane yield was obtained at a hydrothermal reaction temperature of 200°C and decreased at hydrothermal reaction temperatures of 220°C or higher. In particular, this decrease in the methane yield was often reported for the high-temperature hydrothermal pretreatment of diverse biomass. Gossett et al [26] reported that hydrothermal pretreatment could solubilize the cellulose and lignin in the raw material at a hydrothermal reaction temperature of 160°C or higher, but discovered that phenolic substances might be produced, thereby inhibiting the productivity of methanogens. In addition, it has been reported that, as the temperature of the hydrothermal pretreatment increases, the portion of soluble organic matter increases, but the concentration of refractory material in the soluble organic matter also increases [27,28]. Furthermore, Oh and Yoon [29] reported that the methane yields for hydrothermal hydrolysate at 170°C, 180°C, 190°C, 200°C, and 220°C using poultry slaughterhouse wastewater sludge cake were 0.222, 0.242, 0.237, 0.228, and 0.197 Nm^3^/kg-COD_added_, respectively. The lower methane yield at a reaction temperatures higher than 200°C may be attributable to the Maillard reaction in which carbohydrates react with amino acids at high temperatures to form melanoidine with low biodegradability [3,30]. Therefore, the lower methane yield of the ADSC hydrothermal hydrolysate pretreated at the hydrothermal reaction temperature of 220°C in this study may be the effect of recalcitrant substances produced by the cell wall material contained in the sawdust in the ADSC during the high-temperature hydrothermal reaction. Particularly, the ADSC in this study has already undergone anaerobic digestion, and the raw ADSC was composed of difficult-to-decompose organic substances; therefore, the possibility exists that the cellulose, hemicellulose, lignin, etc. contained in the sawdust could be converted into phenolic compounds during the hydrothermal pretreatment. In addition, another possibility is that carbohydrates could react with amino acids at high temperatures according to the Maillard reaction, because ADSC contains large amounts of cellulose and nitrogen sources such as bacterial cells.

## CONCLUSION

In Korea, beef and dairy cattle mainly use sawdust as a bedding material, and the cattle manure is discharged as a sawdust mixture with a moisture content of about 70%. Therefore, a specially designed solid phase anaerobic digestion process is developed for the anaerobic digestion of cattle manure, or the moisture content is to be regulated to keep proper fluidity for the improvement of agitation efficiency in order to input into the conventional type wet anaerobic digester. In particular, sawdust contained in cattle manure has a high lignin content, that is not decomposed well in an anaerobic digester and causes an anaerobic digestion efficiency to decrease. Therefore, in this study, for the wet anaerobic digestion of cattle manure discharged from a sawdust barn, a process for improving the overall anaerobic digestion efficiency was reviewed by the hydrothermal pretreatment of the ADSC and reintroducing ADSC hydrolysate to the anaerobic digester. The hydrothermal pretreatment of the ADSC was performed in a high-temperature and high-pressure closed-type reactor, and the methane yield of the ADSC hydrothermal hydrolysate was the highest at 200°C, 0.112 Nm^3^/kg-VS_added_. However, as a result of analyzing the production of methane that can be recovered from 1 ton of ADSC based on the mass balance, although the methane yield (0.092 Nm^3^/kg-VS_added_) at 180°C was lower than the methane yield (0.112 Nm^3^/kg-VS_added_) at 200°C, methane recovery (18.0 Nm^3^/ton-ADSC) at 180°C was higher than methane recovery (17.4 Nm^3^/ton-ADSC) at 200°C. This is due to the decrease in the content of residual organic matter (VS) by the enhancement of dehydration, carboxylation, and decarboxylation reactions as the hydrothermal pretreatment temperature increases. In particular, hydrothermal pretreatment at 200°C improved the methane yield of ADSC hydrothermal hydrolysate more than that at 180°C, however, the heat capacity corresponding to 200°C requests more consuming energy than the hydrothermal pretreatment reaction at 180°C. Therefore, the hydrothermal reaction condition of 180°C is more economical in terms of the energy efficiency of the hydrothermal pretreatment process. In this study, the overall methane production could be improved through hydrothermal pretreatment of ADSC, nevertheless, the proportion of non-degradable organic matter content showed 76.7% even after hydrothermal pretreatment at 180°C, and a large amount of VS was present in a non-degradable form. This result shows that the efficiency of hydrothermal hydrolyzation of lignin contained in sawdust is low. Therefore, recently, a solid fuel production technique has been developed by the mechanical dehydration of the non-degradable solids generated in the hydrothermal pretreatment of ADSC for the diversifying the utilization of biomass energy. The results of this study suggest the possibility of economical energy conversion of cattle manure, which effectively converts ADSC into energy. However, commercialization of the hydrothermal pretreatment technology would require the process energy efficiency and the recovery of solid fuel by hydrothermal carbonization reaction to be studied in greater detail.

## Figures and Tables

**Table 1:** Chemical composition of inoculum

Parameters	pH (−)	TS (mg/L)	VS (mg/L)	TKN (mg/L)	NH_4_^+^-N (mg/L)	COD_Cr_ (mg/L)	SCOD_Cr_ (mg/L)	Alkalinity (mg/L as CaCO_3_)	TVFAs (mg/L as acetate)
Inoculum	7.91	59,347	31,007	5,188	3,262	39,250	2,498	34,238	1,165

All data means the average value from three replicates (n = 3).

TS, total solid, VS, volatile solid; TKN, total kjeldahl nitrogen; NH_4_^+^-N, ammonium nitrogen; COD_Cr_, chemical oxygen demand; SCOD_Cr_, soluble chemical oxygen demand; TVFAs, total volatile fatty acids.

**Table 2:** Elemental compositions and theoretical methane potential of hydrothermal hydrolysates in the hydrothermal pre-treatment of anaerobic digestion sludge cake

Parameters	ADSC	Hydrothermal reaction temperature (°C)			

		160	180	200	220
Elemental composition (wt. %, d.b.^1)^)					
C	34.0	37.5	37.2	36.4	35.2
H	3.9	4.1	3.9	3.8	3.6
O	30.1	28.1	29.4	25.1	26.7
N	2.3	2.1	1.9	2.3	2.0
S	1.2	1.3	1.1	1.2	1.4
B_th_^2)^ (Nm^3^-CH_4_/kg-VS_added_)	0.425	0.478	0.462	0.496	0.466

All data means the average value from three replicates (n = 3).

ADSC, anaerobic digestion sludge cake.

1) Dry basis.

2) Theoretical methane potential.

**Table 3:** Chemical characteristics of hydrothermal hydrolysates in the hydrothermal pre-treatment of anaerobic digestion sludge cake

Parameters	ADSC	Hydrothermal reaction temperature (°C)				p-value	SEM

		160	180	200	220		
TS (mg/kg)	267,370^a^	260,375^a^	257,419^a^	203,799^c^	227,572^b^	<0.05	6,534
VS (mg/kg)	189,545^a^	181,628^b^	171,833^b^	140,056^c^	145,909^c^	<0.05	5,218
TKN (mg/kg)	11,448^a^	9,577^b^	9,886^b^	8,867^c^	9,759^b^	<0.05	250
NH_4_^+^-N (mg/kg)	6,180^a^	4,430^b^	4,213^c^	4,220^bc^	4,334^bc^	<0.05	205
SCOD_Cr_ (mg/L)	14,430^d^	39,595^c^	43,900^b^	48,215^a^	42,620^b^	<0.05	3,214
COD_s_^1)^ (wt. %, w.b.^2)^)	-	63.56^c^	67.13^b^	70.07^a^	66.14^b^		

All data means the average value from three replicates (n = 3).

ADSC, anaerobic digestion sludge cake; SEM, standard error of the mean; TS, total solid; VS, volatile solid; TKN, total kjeldahl nitrogen; NH_4_^+^-N, ammonium nitrogen; SCOD_Cr_, soluble chemical oxygen demand; COD_s_, chemical oxygen demand solubilization degree.

1) COD solubilization degree of ADSC by the hydrothermal pretreatment.

2) Wet basis.

a–d Mean with different letter differs significantly between treatment (DMRT; Duncan’s multiple range test, p<0.05).

**Table 4:** Methane yield characteristics analysis by the modified Gompertz model in the hydrothermal pretreatment of ADSC

Parameters	ADSC	Hydrothermal reaction temperature (°C)			

		160	180	200	220
B_u_-G (Nm^3^-CH_4_/kg-VS_added_)	0.047±0.003^1)^	0.075±0.001	0.092±0.002	0.112±0.0002	0.104±0.001
R_m_ (mL/d)	2.6±0.1	3.9±0.02	4.9±0.1	5.8±0.03	5.3±0.1
λ^2)^ (d)	2.7±0.1	1.7±0.04	1.6±0.3	1.1±0.1	1.6±0.1
VS_r_^3)^ (wt. %, d.b.^4)^)	10.4±0.6	15.6±0.2	20.6±0.5	23.9±0.04	21.0±0.1
RMSD	0.006	0.008	0.010	0.009	0.010

All data means the average value from three replicates (n = 3).

ADSC, anaerobic digestion sludge cake; B_u_-G, biochemical methane potential by the modified Gompertz model; R_m_, maximum methane production rate; RMSD, root mean square deviation of the modified Gompertz model.

1) Standard deviation.

2) Lag growth phase time.

3) Degree of degradation (B_u_-G/B_th_).

4) Dry basis.

**Table 5:** Methane yield and organic fraction characteristics analysis by the Parallel first-order kinetic model in the hydrothermal pretreatment of ADSC

Parameters	ADSC	Hydrothermal reaction temperature (°C)			

		160	180	200	220
B_u_-P^1)^ (Nm^3^-CH_4_/kg-VS_added_)	0.054±0.003	0.086±0.002	0.105±0.002	0.124±0.001	0.118±0.003
VS_r_^2)^ (wt. %, d.b.^3)^)	11.8±0.7	17.9±0.4	23.3±0.5	26.7±0.2	23.7±0.6
f_e_^4)^	0.066±0.002	0.641±0.007	0.651±0.041	0.669±0.067	0.540±0.024
k_1_^5)^ (1/s)	0.068±0.003	0.094±0.001	0.091±0.007	0.084±0.003	0.089±0.007
k_2_ (1/s)	0.022±0.0004	0.020±0.001	0.022±0.004	0.027±0.011	0.027±0.003
Organic fractions^6)^					
VS_B_^7)^ (wt. %, d.b.)	11.9±0.7	17.9±0.4	23.3±0.5	26.7±0.2	23.7±0.6
VS_e_^8)^ (wt. %, d.b.)	5.5±0.3	11.5±0.1	15.2±0.8	17.8±1.8	12.8±0.3
VS_p_^9)^ (wt. %, d.b.)	6.3±0.4	6.5±0.3	8.1±1.1	8.8±1.8	10.9±0.8
VS_NB_^10)^ (wt. %, d.b.)	88.1±0.7	82.1±0.4	76.7±0.5	73.3±0.2	76.3±0.6
RMSD^11)^	0.001	0.001	0.002	0.003	0.002

All data means the average value from three replicates (n = 3).

ADSC, anerobic digestion sludge cake; VS, volatile solid.

1) Biochemical methane potential by the parallel first-order kinetics model.

2) Degree of degradation (B_u_-P/B_th_).

3) Dry basis.

4) Distribution coefficient of the parallel first-order kinetics model.

5) First-order reaction rate constant.

6) Organic fractions estimated by the parallel first-order kinetics model.

7) Biodegradable volatile solid.

8) Easily biodegradable volatile solid.

9) Persistently biodegradable volatile solid.

10) Non-biodegradable volatile solid.

11) Root mean square deviation of the Parallel first-order kinetic model.

## References

[1] Park M, Kim N, Lee S, Yeon S, Seo JH, Park D (2019). A study of solubilization of sewage sludge by hydrothermal treatment. J Environ Manag.

[2] Carlsson M, Lagerkvist A, Morgan-Sagastume F (2012). The effects of substrate pre-treatment on anaerobic digestion systems: a review. Waste Manag.

[3] Bougrier C, Delgenès JP, Carrère H (2008). Effects of thermal treatments on five different waste activated sludge samples solubilisation, physical properties and anaerobic digestion. Chem Eng J.

[4] Cao Z, Jung D, Olszewski MP, Arauzo PJ, Kruse A (2019). Hydrothermal carbonization of biogas digestate: Effect of digestate origin and process conditions. Waste Manag.

[5] Wirth B, Eberhardt G, Lotze-Campen H Hydrothermal carbonization: influence of plant capacity, feedstock choice and location on product cost.

[6] Ferrer I, Ponsá S, Vázquez F, Font X (2008). Increasing biogas production by thermal (70°C) sludge pre-treatment prior to thermophilic anaerobic digestion. Biochem Eng J.

[7] Oh SY, Kim CH, Yoon YM (2015). The bioenergy conversion characteristics of feedlot manure discharging from beef cattle barn. Korean J Soil Sci Fert.

[8] Ramke HG, Blöhse D, Lehmann HJ, Fettig J, Cossu R, Diaz LF, Stegman R (2009). Hydrothermal carbonization of organic waste. Twelfth International Waste Management and Landfill Symphosium.

[9] Ahring BK, Ibrahim AA, Mladenovska Z (2001). Effect of temperature increase from 55 to 65 degrees C on performance and microbial population dynamics of an anaerobic reactor treating cattle manure. Water Res.

[10] Marin-Batista J, Villamil J, Qaramaleki S, Coronella C, Mohedano A, de La Rubia M (2020). Energy valorization of cow manure by hydrothermal carbonization and anaerobic digestion. Renew Energy.

[11] Omar R, Harun RM, Mohd Ghazi T Anaerobic treatment of cattle manure for biogas production.

[12] Boyle W, Schlegel HG, Barnea J (1977). Energy recovery from sanitary landfills-a review. Microbial energy conversion.

[13] Angelidaki I, Alves M, Bolzonella D (2009). Defining the biomethane potential (BMP) of solid organic wastes and energy crops: a proposed protocol for batch assays. Water Sci Technol.

[14] Lay JJ, Li YY, Noike T (1998). Mathematical model for methane production from landfill bioreactor. J Environ Eng.

[15] Luna-deRisco M, Normak A, Orupõld K (2011). Biochemical methane potential of different organic wastes and energy crops from Estonia. Agron Res.

[16] Rice E, Baird R, Eaton A, Clesceri L, APHA (American Public Health Association) (2012). Standard method for the examination of water and wastewater.

[17] Sørensen AH, Winther-Nielsen M, Ahring BK (1991). Kinetics of lactate, acetate and propionate in unadapted and lactate-adapted thermophilic, anaerobic sewage sludge: the influence of sludge adaptation for start-up of thermophilic UASB-reactors. Appl Microbiol Biotechnol.

[18] Ardİc I, Taner F (2005). Effects of thermal, chemical and thermochemical pretreatments to increase biogas production yield of chicken manure. Fresenius Environ Bull.

[19] Mladenovska Z, Hartmann H, Kvist T, Sales-Cruz M, Gani R, Ahring BK (2006). Thermal pretreatment of the solid fraction of manure: impact on the biogas reactor performance and microbial community. Water Sci Technol.

[20] Yoneyama N, Morimoto H, Ye CX, Ashihara H, Mizuno K, Kato M (2006). Substrate specificity of N-methyltransferase involved in purine alkaloids synthesis is dependent upon one amino acid residue of the enzyme. Mol Genet Genomics.

[21] Kim H, Jeon YW (2015). Effects of hydro-thermal reaction temperature on anaerobic biodegradability of piggery manure hydrolysate. Korean J Soil Sci Fert.

[22] Wang L, Chang Y, Li A (2019). Hydrothermal carbonization for energy-efficient processing of sewage sludge: a review. Renew Sustain Energy Rev.

[23] Funke A, Ziegler F (2010). Hydrothermal carbonization of biomass: a summary and discussion of chemical mechanisms for process engineering. Biofuel Bioprod Biorefin.

[24] Libra JA, Ro KS, Kammann C (2011). Hydrothermal carbonization of biomass residuals: a comparative review of the chemistry, processes and applications of wet and dry pyrolysis. Biofuels.

[25] Kim SH, Kim H, Kim CH (2012). Effect of the pretreatment by thermal hydrolysis on biochemical methane potential of piggery sludge. Korean J Soil Sci Fert.

[26] Gossett RW, Brown DA, Young DR (1981). Predicting the bioaccumulation and toxicity of organic compounds. Coastal Water Research Project Biennial Report.

[27] Gao Y, Liu Y, Zhu G (2018). Microwave-assisted hydrothermal carbonization of dairy manure: Chemical and structural properties of the products. Energy.

[28] Jain S, Sharma M (2011). Power generation from MSW of Haridwar city: a feasibility study. Renew Sustain Energy Rev.

[29] Oh SY, Yoon YM (2017). Energy recovery efficiency of poultry slaughterhouse sludge cake by hydrothermal carbonization. Energies.

[30] Martins SI, Jongen WM, Van Boekel MA (2000). A review of Maillard reaction in food and implications to kinetic modelling. Trends Food Sci Technol.

